# Inaugural Christianson Syndrome Association conference: families meeting for the first time

**DOI:** 10.1186/1866-1955-6-13

**Published:** 2014-05-23

**Authors:** David M Stein, Alan Gerber, Eric M Morrow

**Affiliations:** 1 Department of Molecular Biology, Cell Biology and Biochemistry, Institute for Brain Science, Brown University, Lab for Molecular Medicine, 70 Ship Street, Providence, RI 02912, USA; 2 Department of Psychiatry and Human Behavior, Developmental Disorders Genetics Research Program, Emma Pendleton Bradley Hospital, Alpert Medical School of Brown University, 1011 Veteran Memorial Parkway, East Providence, RI 02915, USA

**Keywords:** Christianson syndrome, Intellectual disability, NHE6, SLC9A6, X-linked developmental disorder

## Abstract

Christianson syndrome (CS) is an X-linked neurodevelopmental disorder caused by deleterious mutations in *SLC9A6*. Affected families organized the inaugural Christianson Syndrome Association conference to advance CS knowledge and develop questions that may be prioritized in future research.

## Introduction

Christianson syndrome (CS) is a novel, X-linked developmental brain disorder, clinically recognized by the symptoms of global developmental delay, intellectual disability, ataxia, epilepsy, non- or minimally verbal status, ophthalmoplegia and most often postnatal microcephaly [1]. CS is caused by deleterious mutations in *SLC9A6*, an X-linked gene that codes for the endosomal Na^+^/H^+^ exchanger 6 (*NHE6*), which is believed to be involved with circuit development [2,3]. Another report has also demonstrated downregulation of *SLC9A6* expression in the autism postmortem brain [4], indicating that the pathophysiology may be related to a subset of autism.

CS may be among the most common X-linked developmental brain disorders. In a large-scale sequencing project in approximately 200 pedigrees with suspected X-linked intellectual disability, two protein-truncating mutations in *SLC9A6* were found [5]. These data place *SLC9A6* among the top six genes with recurrent protein-truncating mutations, and suggest that CS may constitute approximately 1% to 2% of X-linked developmental brain disorders. Assuming between 1% and 3% of the world’s population is diagnosed with an intellectual disability, and approximately 10% to 20% of the causes are due to X-linked genes, CS may affect between one in 16,000 and one in 100,000 people. By comparison, this represents 10% to 50% of the prevalence of Fragile-X syndrome, the most common inherited form of intellectual disability.

### Organization of the meeting

The following is a meeting report of the Christianson Syndrome Association (CSA) Inaugural Conference that took place between June 27th and June 29th, 2013 at the Warren Alpert Medical School of Brown University, Providence, RI, USA. The event was organized by the CSA board members, and was hosted by Dr Eric Morrow of Brown University. Ten families traveled from across the USA, Canada, and Europe to participate, and included boys with CS, their parents, and their unaffected siblings.

CSA was founded in 2011 to impart a sustained vision of hope for the future of patients with CS and their families through advancing awareness and treatment. The conference slogan, ‘A New Day Has Come’, reflects the confidence that increasing awareness will bring exciting new research and treatment [6].

### Christianson Syndrome Association inaugural conference

The CSA symposium began on the evening of Thursday 27 June, as families were welcomed by board members and invited to a ‘meet and greet’ event. This was an exciting experience because most of the families had never encountered another individual affected by CS and because the boys were able to meet each other.

On Friday 28 June, the first full day began with a welcome from Deborah Nash, president of CSA. Nash reviewed the history and mission of CSA and introduced its board members. She described her initial feelings of helplessness when her child was diagnosed with CS, as no pre-existing information had been available to help her. Motivated by the early experiences with her son, she founded the CSA in 2011. Nash concluded with the CSA mission: ‘to advance the awareness and treatment of CS through education and information, research, advocacy, and support for individuals with CS, their families, and other concerned parties’.

Friday’s main speaker was Dr Eric Morrow (Brown University, USA) who presented his laboratory’s research on CS (all of which was unpublished) and conducted a question and answer session with the families. Many of the families who had participated in Morrow’s research were eager to discuss the early results. Morrow described three types of research studies in his laboratory with regard to CS. The first study is an analysis of the clinical symptoms and developmental trajectories of boys affected by CS. The goal of this ongoing study is to teach families what to expect when their son is first diagnosed and to guide future treatments of CS. Morrow presented results from a soon-to-be-published study on 15 prospectively recruited families, with affected patients ranging in age from 2 to 22 years. Among the important new findings was the high rate of regressions seen in over 40% of patients, ranging in age of regression from 15 months to 16 years (unpublished data). The second study aims to develop mouse models for understanding CS pathophysiology. Morrow discussed these current projects on mice with *SLC9A6* mutations, including a study that demonstrates increased endosome acidity in mutant neurons [3]. Morrow also reviewed a previously published mouse study wherein histopathology consistent with endo-lysosomal disease was seen in the hippocampus, cortex, amygdala, and cerebellum [7]. Morrow reviewed a final study from his own laboratory, which examines induced pluripotent stem cells from patients with CS. The objective of this work is to investigate the neurogenetic underpinnings of CS in human neuronal cells, and to develop a patient-derived cellular system for drug screening. Throughout his talk, Morrow emphasized the motto ‘research equals hope’ for CS patients and their families.

For Friday’s second and third sessions, the families were divided into two groups. Each group alternated between hearing from Nicole Ener (Magnolia West High School, USA), a teacher with considerable experience working with CS, and taking a tour of the research facilities in Morrow’s laboratory. The boys affected by CS played together in an adjacent room with CSA-provided care-takers and their unaffected siblings during these sessions.

Ener has taught special education in the school system and has specifically worked with a CS patient for several years. She guided a discussion of home and school interventions that would benefit children with CS. She also provided principal suggestions for integrating children with CS into a lesson plan. To ensure children with CS maintain focus, she stressed the importance of minimizing distractions. In order to optimize lessons, short intense instruction is required, followed by a quick break. Educators are encouraged to rotate often when working with a child affected by CS because this approach to teaching can be tiring. Additionally, people with CS tend to engage in a high degree of oral stimulation so this needs to be utilized, instead of challenged, when planning lessons. Finally, boys with CS must stay physically active and should be encouraged to use their motor skills as often as possible. Ener concluded by suggesting that raising a child with CS takes a whole team, and that parents need to be vigilant about communication with all staff involved, especially teachers and therapists.

In Friday’s final session, Morrow led a discussion to determine the greatest problems each family encountered, and to agree on the lead research questions. In a collaborative dialogue, families in attendance concurred on a number of first-line questions (Table 1). The first major question presented by the families was: What are optimal treatments for co-occurring epilepsy that affects all CS patients? The families voiced a possibility of conducting treatment trials to determine efficacious anti-epileptic therapies in CS. The second question was: What is the predicted life expectancy for boys currently affected with CS, and what is their anticipated quality of life as they mature during puberty and into adulthood? The parents were concerned with the undetermined clinical features of CS beyond the school-age years. In Christianson’s first report, from an analysis of a single large pedigree with CS in South Africa, the article introduced the possibility that there may be a risk of early mortality in CS [1]. Subsequent studies have also raised the possibility of a neurodegenerative component in CS [8]. The parents were eager to meet more families with older CS patients, as the oldest CS conference participant at the meeting was 17 years old. The final question posed by families concerned the symptoms of CS that are not as commonly reported. The focus was to understand why these symptoms were present in their children and to elucidate other observed symptoms that may be related to CS.

**Table 1:** This table outlines the major research questions that were designed in a collaborative discussion between the executive board of the Christianson Syndrome Association and parents of patients with Christianson Syndrome

	**Priority questions proposed by Christianson Syndrome families for research inquiry**
1.	What are optimal treatments for co-occurring epilepsy with Christianson Syndrome?
2.	To what extent is Christianson Syndrome progressive? What is the predicted life expectancy for boys currently affected with Christianson Syndrome?
3.	What are the less-known symptoms in Christianson Syndrome, and why are they present in the boys?

The event concluded on Saturday 29 June with a session for parents and siblings, where the future goals of CSA were discussed. This session included several considerations for the CSA’s expansion. The parents established that maintaining a network of support for CS patients and their families was a crucial pillar of the organization, and suggested further events for fundraising and awareness. The second goal was presented by the executive board, and determined that continued financial contribution for CS research should be a major objective. Further, Morrow agreed to design a day-long scientific meeting at the next CSA meeting with the larger community of clinicians and researchers with an interest in CS and/or relevant expertise regarding the critical problems presented by CS. Researchers and clinicians with interest in participating are encouraged to contact Dr. Morrow to communicate their ideas and interest (eric_morrow@brown.edu). The final goal aims to expand the CSA, particularly in international locations throughout Canada and Europe, in the hope of attracting more families to the organization’s collaborative benefits.

## Conclusion

This inaugural conference was a major step made to unite boys, families, care-givers, CSA staff, and researchers who work for CS. At the meeting’s close, the attendees envisioned a future of confidence and solidarity for CSA, as their parting words were shared: ‘We came as strangers, and left as a family’.

## Competing interests

Dr. Morrow discloses that his laboratory has received funding for his research from the Christianson Syndrome Association.

## Authors’ contributions

All three authors (DS, AG, and EM) aided in the organization and implementation of the Christianson Syndrome Association inaugural conference. DS was responsible for performing the majority of writing and editing tasks for this meeting report, while AG and EM contributed and reviewed additional sections. All authors read and approved the final manuscript.

## Authors’ information

DS is a post-baccalaureate research assistant in the Morrow Laboratory who conducts pedigree analyses and wet-lab projects investigating the neurobiological pathways of CS. AG is a clinical research assistant at Bradley Hospital who works on early detection and intervention in children at risk for developmental delay.

EM is a principal investigator at Brown University who explores the genetic and molecular mechanisms underlying disorders of cognitive development, such as intellectual disability and autism, with a specific attention to CS.
